# How Social Reactions to Alcohol-Related Facial Flushing Are Affected by Gender, Relationship, and Drinking Purposes: Implications for Education to Reduce Aerodigestive Cancer Risks

**DOI:** 10.3390/ijerph14060622

**Published:** 2017-06-09

**Authors:** Ian M. Newman, Lanyan Ding, Duane F. Shell, Lida Lin

**Affiliations:** 1 Nebraska Prevention Center for Alcohol and Drug Abuse, Department of Educational Psychology, University of Nebraska-Lincoln, Lincoln, NE 68588, USA; dinglanyan@gmail.com (L.D.); dshell2@unl.edu (D.F.S.); 2 Department of Psychology in Education, School of Education, University of Pittsburgh, Pittsburgh, PA 15260, USA; lil85@pitt.edu

**Keywords:** alcohol, drinking, ALDH, ALD, flushing, university students, China

## Abstract

Alcohol-related facial flushing is a sign of compromised alcohol metabolism and increased risk of certain cancers. This project examined how facial flushing might be used to reduce alcohol use to lower cancer risks. Interviews with Chinese university students identified gender, friendship, and drinking purpose as important variables related to whether someone would encourage a person who flushes when drinking alcohol to stop or reduce their drinking. A questionnaire was developed that incorporated these variables into 24 drinking scenarios in which someone flushed while drinking. Students responded whether they would (a) encourage the flusher to stop or drink less; (b) do nothing while wishing they could; or (c) do nothing because there was no need. Analysis of survey responses from 2912 university students showed a three-way interaction of the variables and implied that the probability students will intervene when a drinker flushes was highest when the flusher was a female, a close friend, and the drinking purpose was for fun and lowest if the flusher was a male, the friendship was general, and the drinking purpose was risky. The results provide important details about the social factors affecting how other people respond to a person who flushes when drinking alcohol. This information is useful for those considering ways to reduce and prevent aerodigestive cancers through education and information programs.

## 1. Introduction

Alcohol (ethanol) has been identified as a causative agent for aerodigestive tract cancers [1,2]. Aerodigestive cancers include cancers of the respiratory tract and the upper part of the digestive tract (lips, mouth, tongue, nose, throat, vocal cords, and part of the esophagus and trachea) [3]. Not all alcohol drinkers are equally at risk. The variability of risk of developing cancer from exposure to alcohol results from individual drinking patterns, drinking in combination with other behaviors like smoking, drinking in combination with environmental exposures, and an inherited deficiency in alcohol metabolism. One evidence of the genetically inherited deficient metabolism of alcohol is a person’s face and neck flushing red when he/she drinks alcohol. Flushing is important because it is perhaps the only clearly visible physiological sign of heightened cancer risk associated with alcohol use. Being visible, facial flushing invites reactions from other people that can moderate the degree of risk. Whether or not other people in the flushing person’s drinking group encourage or discourage further drinking is important. This paper explores the effects of social factors on how other people respond to someone’s visibly flushing.

### 1.1. Physiological Cause of Alcohol-Related Flushing and Cancer Risk

Alcohol (ethanol) metabolism proceeds in two stages. In the first stage, alcohol is metabolized by the enzyme alcohol dehydrogenase (ADH) into acetaldehyde. Acetaldehyde is carcinogenic and toxic, and, in some people, stimulates a histamine reaction visible as a red flush on the face and neck. In the second stage, the aldehyde dehydrogenase (ALDH) isozymes metabolize acetaldehyde into relatively harmless acetic acid that is expelled from the body mostly in the urine. Genetic code for ALDH consists of 19 genes in 11 families [4]. One group, ALDH2, is mainly responsible for acetaldehyde metabolism. Some individuals inherit a mutated form that is enzymatically inactive (ALDH2*2) leading to increased exposure to acetaldehyde in these individuals when ingesting alcohol [5]. Lachenmeier and Salaspuro described a causal relationship between acetaldehyde exposure and upper aerodigestive cancers and suggest the carcinogenicity of acetaldehyde may be higher than previously estimated [6].

One ALDH gene is inherited from each parent. Individuals who inherit two ALDH2*2 genes (homozygous) are essentially unable to metabolize alcohol, experience extreme reactions from the accumulation of acetaldehyde, and as a consequence do not drink or rarely drink. Heterozygous individuals (ALDH2*2/ALDH2*1) experience a greatly reduced ability to metabolize acetaldehyde—about 18% of normal metabolism [7]. In addition to flushing, these individuals also sometimes experience tachycardia, headache, heart palpitation, shortness of breath, hyperventilation, low blood pressure, vertigo, nausea, and vomiting [8] when they drink alcohol. Such uncomfortable physiological responses to alcohol have been suggested as a reason Asian populations consume less alcohol and have fewer alcohol-dependent persons [9]. However, despite the associated discomforts, these individuals often drink through their discomfort and develop some level of tolerance for alcohol. A study by Parrish et al. explored whether social embarrassment from facial flushing could reduce an individual’s exposure to alcohol, but found that some individuals overcome embarrassment from flushing to continue drinking [10]. Observers of flushing drinkers suggest they continue drinking as a result of social pressures and traditions [5,10,11,12,13]. It is the individuals who flush and keep on drinking who have significantly increased risks for upper aerodigestive cancers.

Data from alcoholics in Japan [14] suggest that the number of individual heterozygotes who drink alcohol is increasing: 3% in 1979, 8% in 1986, 13% in 1992. Later data suggests as many as 26% of males in Tokyo drinking more than 400 g of alcohol per week were heterozygotes [15]. If the same trend observed in Japan is occurring in China, combined with evidence that the number of drinkers and the per-capita alcohol use are increasing in China [16,17,18], then there is an increasing risk of upper aerodigestive cancers. Currently in China, among men esophageal cancer (one of the cancers grouped under aerodigestive cancers) is the fourth leading cause of cancer deaths; among women, it is the eighth [19]. It is estimated that half of Chinese, Japanese, Korean, and other Asian people have the genetic predisposition to flush [3]. Brooks et al. estimate that 540 million individuals worldwide are ALDH2-deficient (8%) [20]. A reduction in alcohol consumption among ALDH2-deficient humans could lead to reductions in the occurrence of aerodigestive cancers.

A reduction in alcohol consumption among ALDH-deficient individuals can be achieved if the individuals who flush change their drinking behavior and if those who encourage those individuals to drink more change theirs.

### 1.2. Study Purpose

The purpose of this study was to identify and examine the interaction of social variables that could affect whether a person was willing to suggest to someone who flushes that he/she stop or reduce drinking.

## 2. Materials and Methods

### 2.1. Questionnaire Development

To develop the questionnaire, we initially conducted semi-structured personal interviews with students to identify what students thought were the factors influencing their reaction to a fellow student flushing. In all, 24 Chinese university students participated in the interviews.

Data from the interviews identified three factors that were used for questionnaire development. First, students confirmed the importance of gender as a major predictor of how other people would react to someone’s flushing that was identified in prior studies [21,22,23]. Second, students indicated that people’s reactions to someone’s flushing would depend on whether the person who flushed was a haopengyou (好朋友) or a putongpengyou (普通朋友), which we have translated to English as “a close friend” or “a general friend.” Third, students indicated that two contrasting purposes of the drinking would affect reactions. A fun/pleasure drinking occasion was described as a relaxed drinking environment that served as a means to enjoy the pleasures of drinking and friendship. In contrast, at risky drinking occasions, some drinkers come for the purpose of getting drunk or getting others drunk. These three factors were used to develop the questionnaire for this study.

Because of the contextual nature of reactions reported by students, the questionnaire was designed with a scenario format. Six drinking scenarios were developed as described in Table 1. Three of the drinking scenarios described a drinking occasion to which most of the people came with the purpose of having a fun/pleasurable time with friends: (1) having a leisurely dinner with friends; (2) taking a break during a group work project; and (3) having dinner with a professor after finishing a large project. Three of the drinking scenarios described occasions where the students came together expecting a considerable amount of drinking and the likelihood of getting drunk themselves or getting someone else drunk: (4) toasting at a friend’s birthday party; (5) drinking with senior students at a club dinner; and (6) a graduation party. For each of these six scenarios, there were separate questions about reactions to a male flusher and a female flusher. For each of the scenarios, the participants were given three responses: (1) encourage the flusher to reduce or stop drinking; (2) not encourage the flusher to reduce or stop drinking but felt they should; and (3) not do anything because there was no need to. Each scenario included four possible types of flushers: a close male friend, a general male friend, a close female friend, and a general female friend. This resulted in a 6 × 2 × 2 = 24 design in which all students who took the survey responded to 24 questions (Table 1). Additionally, a question taken from the College Students’ Health Behavior Survey [24] asked whether students had ever observed someone flushing.

### 2.2. Survey Participants

A total of 2912 students from universities in Southwestern, Central, and Northeastern China completed the survey. Data from 2492 (85.6%) of the surveys presented valid results (1114 males; 1378 females). The description of the sample is provided in Table 2.

### 2.3. Data Analysis Plan

A generalized logit mixed model was estimated in Mplus Version 7.0 [25] to evaluate the main and interactive effects of gender, degree of friendship, and drinking purpose on students’ reactions to a person flushing from alcohol use. The scenarios had three possible reactions to facial flushing. These were dummy coded into a multinomial outcome variable for the logistic regression using “not do anything because there was no need to” as the reference category. Generalized modeling was necessary to account for the nominal natural of the outcome, whereas mixed modeling was necessary to account for the dependency among observations due to the nesting of responses within students. Level 1 (responses level) in this model had six fixed effects predictors: gender (0 = male, 1 = female), friendship (0 = general friendship, 1 = close friendship), and scenario indicating drinking purpose (0 = risky, 1 = fun). These predictors have three two-way interactions (scenario × gender, scenario × friendship, and gender × friendship), and one three-way interaction (scenario × gender × friendship). Level 2 (students level) in this model accounted for individual differences in the dependency of responses at Level 1 because one student may provide responses more similar to all questions than another. Level 2 (students level) in this model had random effects for these differences in students’ responses. The complete model is shown in Appendix A.

Full information maximum likelihood with robust variance estimation was used throughout. Likelihood ratio tests (LRTs) were performed to evaluate the omnibus effect of each predictor on the overall outcome (three response options corresponding to two degrees of freedom), and Wald tests were used to determine the significance for the specific comparisons (two response options corresponding to one degree of freedom). The odds ratios were computed to facilitate interpretation.

### 2.4. Data Availability and Ethical Considerations

The survey data upon which the results are based can be accessed at University of Nebraska-Lincoln Data Repository [26]. Responsible authorities at each university approved the project and the surveys. The University of Nebraska-Lincoln Institutional Research Board approved both the qualitative and quantitative procedures prior to the study (Interviews: IRB #20100610932EX; Survey: IRB #20110500663EP).

## 3. Results

The primary purpose of this study was to determine what factors influenced college student responses to someone’s facial flushing. Specifically, the study objective was to determine how gender, drinking setting/purpose, and friendship influenced how students reacted.

The results from the test of the full model with all interactions is shown in Table 3. The odds of suggesting that the flusher stop or reduce drinking versus making no such suggestion were higher with respect to females, close friends, and fun drinking occasions. The three-way interaction among these factors was significant (Table 3). The variance of Level 2 random effects of student on responses for “would suggest the person stop or reduce drinking” versus no suggestion was τ*_suggest_*_,00_ = 3.721, *p* < 0.001, indicating a significant variability in response among students. Similarly, the variance of Level 2 random effects of students on responses for “want to suggest the person stop or reduce drinking, but did not” versus no suggestion was τ*_want_*_,00_ = 2.577, *p* < 0.001, indicating a significant between-student variance in the outcome.

In the follow-up test, holding constant the random effect, the three-way interactive logistic model implied that the probability of suggesting to a flusher to stop or reduce drinking versus not making such a suggestion was the highest when the flushing student was a close female friend in a fun drinking situation and lowest if the flusher was a general male friend in a risky drinking situation.

$$\begin{array}{l} \pi_{Male*Close*Fun}=0.90,{\;\pi }_{Male*General*Fun}=0.72,{\;\pi }_{Female*Close*Fun}\\ \;=0.943,{\;\pi }_{Female*General*Fun}=0.941,{\;\pi }_{Male*\mathrm{Cc}lose*Risky}\\ \;=0.75,{\;\pi }_{Male*General*Risky}=0.44,{\;\pi }_{Female*Close*Risky}\\ \;=0.89,{\;\pi }_{Female*General*Risky}=0.81). \end{array}$$

The odds of wanting to suggest that the flusher stop but not actually doing so versus having no such inclination were lower with respect to female flushers and close friends and were also lower with respect to close friends (of either gender) in fun drinking occasions. The three-way interaction among these factors was not significant (Table 3). However, there were significant two-way interactions for friendship × purpose and gender × friendship, but no significant interactions for gender × purpose.

In the follow-up test, holding constant the type of drinking purpose and the random effect, the two-way interactive logistic model suggested that the probability that a student did not suggest that a flusher stop or reduce but wanted to versus the probability that a student had no such inclination was the highest when the flushing student was a general female friend and the lowest if the flusher was a close male friend.

$$\pi_{male*close}=0.61,{\;\pi }_{male*general}=0.75,{\;\pi }_{female*close}=0.71,{\;\pi }_{female*general}=0.84).$$

When holding constant gender and the random effect, the probability of wanting to make a suggestion versus having no such inclination was highest when the flushing student was a general friend in a fun drinking situation and lowest when the flusher was a close friend in a risky drinking purpose.

$$\pi_{close*fun}=0.71,{\;\pi }_{close*risky}=0.70,{\;\pi }_{general*fun}=0.85,{\;\pi }_{general*risky}=0.76).$$

## 4. Discussion

Alcohol-induced facial flushing is common in Asian populations and indicates in a given individual a compromised ability to metabolize alcohol and an increased risk for upper aerodigestive cancers. When someone’s heightened risk is visible to other people in a drinking situation, it raises questions about how facial flushing could be used as a cue for people to make a suggestion to the person who flushes to reduce or stop their drinking. This study explored the effects of three social factors—gender, degree of friendship, and drinking purpose—on the likelihood that someone would intervene. The results from this study can benefit the development of educational programs aimed at increasing the number of people who reduce or stop drinking because they flush and who lend support to drinking companions who want to stop drinking or slow down. Chang et al. [27] have recently suggested education as an option to reduce aerodigestive cancers.

Almost all of the students in the sample (99%) reported that they regularly or occasionally observed someone flushing when drinking alcohol, leading us to believe that an educational program for Chinese young adults developed around the flushing phenomenon would be relevant.

Our results indicated that the probability that someone would suggest to a flusher that she or he stop or reduce drinking was highest when the flusher was a female and a close friend and when the drinking situation was principally for pleasure. The probability that someone would not intervene even while thinking the flushing person should stop drinking (“bystanders”) was highest either when the flusher was a general female friend or when the flusher was a general friend (regardless of gender) in a fun drinking situation.

Finding that the probability that people will suggest to a person who flushes that they reduce or stop drinking is highest when the person flushing is a female and a close friend and when the drinking situation is for pleasure gives rise to the most likely scenario about which to begin education to modify drinking behaviors.

An educational approach making use of these findings will have to be sensitive to the Chinese cultural context of gender, friendship, and drinking purposes. Specific implications of using each social factor for educational program development are discussed in the following sections.

### 4.1. Gender 

Earlier studies suggested that being a female flusher provided a degree of protection from pressure to drink [21,22,23]. Study findings continue to support that the probability of intervening is higher for female flushers relative to male flushers. However, our new data suggest that the degree of protection is moderated by friendship and drinking purpose. This was especially apparent for males, and the probability of someone suggesting stopping or reducing drinking increased dramatically if it involved a close friendship and the drinking purpose was for fun/pleasure. For females, there were similar increases in the probability of intervening for close friendship and fun/pleasure drinking purposes, but friendship and drinking purpose made less of a difference. Results suggest that, for females, being female is the main criterion others use to intervene across all situations. For males, however, intervention is heavily influenced by friendship and setting. These results suggest that it would be easier to encourage, and to reinforce suggestions to encourage, females who are flushing, compared to males who are flushing, to reduce or stop drinking when drinking took place with close friends in a fun/pleasure drinking situation. Making a flusher aware of this, and suggesting that they seek assistance from close friends and that they avoid risky drinking situations when possible, could be advised.

For bystanders (the students who said they would not intervene but think they should), gender interactions were less pronounced. Again, there was a higher probability that students think they should intervene when the flusher was a female. However, this was not affected by drinking purpose. The probability of a student saying they want to intervene but did not was higher for general rather than close friends for both females and males. This is likely opposite to the probabilities for intervening because students were more likely to actually intervene with a close friend rather than standing by. These results are not necessarily surprising, but given the almost universal acknowledgment by Chinese people that their drinking patterns are deeply culturally ingrained and would be extremely difficult to change, some education on this issue is thus suggested.

### 4.2. Friendship

The students we interviewed said they thought people’s reactions to someone flushing would depend on whether the person who flushed was a haopengyou (好朋友) or a putongpengyou (普通朋友), which we have translated to English as “a close friend” or “a general friend.” Students said that the wish to help a close friend is always balanced against the status roles and drinking etiquette in a drinking situation, so while a person may wish to help out a close friend, they may not be able to take action to intervene. For example, drinking at an official banquet with bosses or dignitaries is a situation where status roles and rules of etiquette prevail over individual wishes. Students believed that, while the degree of friendship could not change the rules of etiquette, it might change the degree to which a person followed the rules.

The study results strongly supported the interaction of friendship with the drinking setting, as well as with the gender of the flusher. This was especially true for males more so than females. The probabilities of intervention were much higher when the male was a close friend, whereas they were similar for females regardless of the closeness of the friendship. Similarly, bystanding (being a person who does not intervene, but thinks the flusher should reduce or stop drinking) was more likely for general friends of either gender. This suggests hesitancy to speak up or take action when the flusher is only a casual acquaintance.

### 4.3. Drinking Purpose

Students we interviewed described contrasting types of occasions where drinking together would occur, which we have classified as fun/pleasure occasions and risky occasions. A fun/pleasure drinking occasion was described as a relaxed drinking environment that served as a means to enjoy the pleasures of drinking and friendship. People at fun/pleasure drinking occasions would not necessarily refuse to get drunk, but getting drunk was not the priority. With fun/pleasure as the unspoken goal, anything that might detract from the fun or pleasure of the occasion would be noticed and resisted. For instance, a person’s flushing while drinking could be interpreted as a sign of that person’s discomfort, and by extension a threat to the pleasantness of the occasion, so people in the group might encourage the flusher to stop or drink less.

In contrast, at risky drinking occasions, some drinkers come with the purpose of getting drunk or getting others drunk. If the majority of the people in the group shared this objective, an individual’s wishes were easily overridden. Anyone who appeared to get in the way of the group’s objective may become the focus of the group’s attention. If someone in the group flushed and flushing was seen as an obstacle to the group’s intentions, members of the group could either ignore the flusher or informally work together to get the flusher drunk. The group’s dynamics in these risky drinking occasions may mean that flushers will fight to overcome the physical discomfort of their reaction to alcohol and get drunk. In risky situations, someone who flushes might also pressure other people to drink in order to get others to share in the discomfort and to redirect attention away from themselves.

The study results thus support the importance of the drinking setting and purpose for taking action. It was always the case that students were more likely to intervene in a fun setting than in a risky setting. This was true across both gender and friendship status. However, the effect of the setting was greater for males than females, as the probability of intervening was much lower for males in risky drinking settings. For bystanders, however, the setting made little difference. Setting did not interact with gender, and the probability of not suggesting but wanting to was only marginally higher for general friends in risky drinking situations. These findings suggest that the effect of drinking setting/purpose on the probability of taking overt action is much stronger than that on the probability of thinking that perhaps they should do something but did not.

### 4.4. Limitations

These results need to be viewed in the context of several limitations. Our results are based on scenarios representing hypothetical situations. Reactions to hypothetical situations may not reflect actual reactions to actual situations. The identification of gender, degree of friendship, and drinking purpose used in this questionnaire was based on interviews from students at only one university. Moreover, while the sample was students from 13 universities in different parts of the country, we cannot claim it is truly representative of all undergraduate university students in China, nor is it representative of China’s general population. Interpreting the results from written questionnaires is always dependent upon the honesty of the respondents. The sample was intentionally homogeneous—undergraduate university students—limiting extrapolation of the results to other populations.

## 5. Conclusions

New evidence linking alcohol-induced facial flushing to increased aerodigestive cancer risk creates a need to develop prevention and education. This study suggests the effects of social factors on the likelihood that individuals will encourage someone who flushes to stop or reduce drinking. This study identified social factors other than gender, which had been identified in earlier studies, namely the degree of friendship and the drinking purpose, that may increase the likelihood of a person’s suggesting that someone who flushes should stop or reduce drinking.

## Figures and Tables

**Table 1:** Matrix illustrating question logic.

Scenario	Gender	Friendship	What Would You Do?
Six scenarios for drinking purpose: three for fun/pleasurable drinking; three for risky drinking	If the friend who flushed is a male,	and if you and he are good friends,	(1) I would suggest that he stop drinking or drinking less.
			(2) I would not suggest that he stop drinking or drinking less, even though I think he should.
			(3) I would not suggest that he stop drinking or drinking less, bacause I do not think he needs to.
		and if you and he are general friends,	(1) I would suggest that he stop drinking or drinking less.
			(2) I would not suggest that he stop drinking or drinking less, even though I think he should.
			(3) I would not suggest that he stop drinking or drinking less, bacause I do not think he needs to.
	If the friend who flushed is a female,	and if you and she are good friends,	(1) I would suggest that she stop drinking or drinking less.
			(2) I would not suggest that she stop drinking or drinking less, even though I think she should.
			(3) I would not suggest that she stop drinking or drinking less, bacause I do not think she needs to.
		and if you and she are general friends,	(1) I would suggest that she stop drinking or drinking less.
			(2) I would not suggest that she stop drinking or drinking less, even though I think she should.
			(3) I would not suggest that she stop drinking or drinking less, bacause I do not think she needs to.

**Table 2:** Sample age, year in university, and flushing experience.

Respondent Characteristics		Male		Female		Total	
		*n*	%	*n*	%	*n*	%
Demographics							
Age	18 and under	76	6.8	136	9.9	212	8.5
	19 and 20	611	54.8	706	51.2	1317	52.8
	21 and older	427	38.4	536	38.9	963	38.7
Year	Freshman	679	61.0	686	49.8	1365	54.8
	Sophomore	178	16.0	258	18.7	436	17.5
	Junior	137	12.2	297	21.6	434	17.4
	Senior	120	10.8	137	9.9	257	10.3
Flushing							
Observed others’ flushing?	Frequently	782	70.2	982	71.3	1764	70.8
	Occasionally	317	28.5	383	27.8	700	28.2
	Never	15	1.3	13	0.9	28	1.0
	Total	1114	100	1378	100	2492	100

**Table 3:** Logistic regression results for reactions to someone’s alcohol-related facial flushing.

Variables	$\hat{\gamma }$	$\hat{SE}$	$\hat{OR}$
Would suggest stop/reduce vs. no			
Gender	1.670 ***	0.058	5.31
Friendship	1.339 ***	0.054	3.817
Drinking purpose	1.179 ***	0.054	3.252
G × F	−0.641 ***	0.058	0.527
G × P	0.159 ***	0.062	1.172
F × P	−0.059	0.051	0.943
G × F × P	0.402 ***	0.080	1.495
Want to suggest stop/reduce vs. no			
Gender	0.620 ***	0.049	1.859
Friendship	−0.529 ***	0.051	0.589
Drinking purpose	0.589 ***	0.047	1.803
G × F	−0.320 ***	0.061	0.726
G × P	0.081	0.064	1.084
F × P	−0.299 ***	0.057	0.741
G × F × P	0.254	0.089	1.289

*** *p* < 0.001. 0 = male, 1 = female. 0 = general friend, 1 = close friend. 0 = risky drinking purpose, 1 = fun drinking purpose. OR = odds ratio. G = gender, P = drinking purpose, F = friendship, G × F = gender × friendship, G × P = gender × drinking purpose, F × P = friendship × drinking purpose.

## Appendix A

The equations for the generalized logit mixed mode are as follows:

(A1) $$\begin{array}{l} \text{log}\left(\frac{\pi_{s,ij}}{\pi_{nd,ij}}\right)=\gamma_{s,00}+\gamma_{s,10}{\text{Gender}}_{ij}+\gamma_{s,20}{\text{Friend}}_{ij}+\gamma_{s,30}{\text{Scenario}}_{ij}\\ \;+\;\gamma_{s,40}{\text{Gender}}_{ij}\times {\text{Friend}}_{ij}+\gamma_{s,50}{\text{Gender}}_{ij}\times {\text{Scenario}}_{ij}\\ \;+\gamma_{s,60}{\text{Friend}}_{ij}\times {\text{Scenario}}_{ij}+\;\gamma_{s,70}{\text{Gender}}_{ij}\times {\text{Friend}}_{ij}\\ \;\times {\text{Scenario}}_{ij}+\;\gamma_{s,70}{\text{Gender}}_{ij}\times {\text{Friend}}_{ij}\times {\text{Scenario}}_{ij}+u_{s,0j} \end{array}$$

(A2) $$\begin{array}{l} \text{log}\left(\frac{\pi_{ds,ij}}{\pi_{nd,ij}}\right)=\gamma_{ds,00}+\gamma_{s,10}{\text{Gender}}_{ij}+\gamma_{ds,20}{\text{Friend}}_{ij}+\gamma_{ds,30}{\text{Scenario}}_{ij}\\ \;+\;\gamma_{ds,40}{\text{Gender}}_{ij}\times {\text{Friend}}_{ij}+\gamma_{ds,50}{\text{Gender}}_{ij}\times {\text{Scenario}}_{ij}\\ \;+\gamma_{ds,60}{\text{Friend}}_{ij}\times {\text{Scenario}}_{ij}+\;\gamma_{ds,70}{\text{Gender}}_{ij}\times {\text{Friend}}_{ij}\\ \;\times {\text{Scenario}}_{ij}\;+u_{ds,0j} \end{array}$$

where $\log\left(\frac{\pi_{nd,ij}}{\pi_{s,ij}}\right)$ is the log odds of a suggestion versus no desire to suggest (and no suggestion) by the jth response to the ith item; $\log\left(\frac{\pi_{ds,ij}}{\pi_{s,ij}}\right)$ is the log odds of a desire to suggest (but no suggestion) versus no desire to suggest (and no suggestion); $\gamma_{s,00}\ldots \gamma_{s,70}$ and $\gamma_{ds,00}\ldots \gamma_{ds,70}$ are the respective fixed effects for each log odds comparison; $u_{s,0j}$ and $u_{ds,0j}$ are the respective random effects under the assumption $u_{s,0j}\sim N\left(0,\tau_{s,00}\right)$ and $u_{ds,0j}\sim N\left(0,\tau_{ds,00}\right)$; all predictor variables are dummy coded with reference groups corresponding to males (0 = male, 1 = female), close friends (0 = general, 1 = close), and fun scenarios (0 = risky, 1 = fun).
